# Factors associated with offer and uptake of provider-initiated HIV testing and counselling among men attending healthcare facilities in Moshi Municipality, Northern Tanzania

**DOI:** 10.1371/journal.pone.0291792

**Published:** 2023-09-20

**Authors:** Hellen Kyilyosudu, Sia E. Msuya, James S. Ngocho, Damian J. Damian

**Affiliations:** 1 Institute of Public Health, Department of Community Health, Kilimanjaro Christian Medical University College (KCMUCo), Moshi, Tanzania; 2 St. Joseph Council Designated Hospital, Moshi, Tanzania; 3 Institute of Public Health, Department of Epidemiology & Biostatistics, Kilimanjaro Christian Medical University College (KCMUCo), Moshi, Tanzania; 4 Department of Community Medicine, Kilimanjaro Christian Medical Centre (KCMC), Moshi, Tanzania; South African Medical Research Council (SAMRC) / Stellenbosch University (SU), SOUTH AFRICA

## Abstract

**Background:**

HIV Testing and Counseling is a critical entry-point for HIV care, treatment and prevention. Tanzania adopted the WHO recommendations of Provider-Initiated HIV Testing and Counseling (PITC) in 2007 with the aim of increasing early HIV diagnosis and timely access to treatment and support services. However, approximately 55% of men are still unaware of their HIV status. This study aimed to determine the level of PITC uptake and factors associated with PITC availability and uptake among men attending healthcare facilities in Moshi Municipality, Northern Tanzania.

**Method:**

A facility-based cross-sectional study was conducted in July 2019 in five selected healthcare facilities in Moshi Municipal, Kilimanjaro region. Exit interviews were conducted with men aged 18 years and above who attended for care in the selected facilities. Modified Poisson regression modelling with robust standard errors were used to determine factors independently associated with being offered and accepting the PITC offer.

**Results:**

A total of 562 men participated in this study. The median age of participants at enrollment was 37 (IQR: 26–59) years. Only 58% of participants reported to have been offered provider-initiated HIV counseling. Of these, 83% accepted the offer of HIV testing. Age between 35–59 years (aPR = 1.2; 95% Confidence Interval (CI): 1.0, 1.4; *p* = 0.033) and having primary education (aPR = 0.7; 95% CI: 0.6, 0.9; *p* = 0.010) were factors independently associated with being offered PITC. Age between 35–59 years (aPR = 0.8; 95% CI: 0.7, 0.9; *p* = 0.002); having been previously tested for HIV (aPR = 1.3; 95% CI: 1.1, 1.5; *p* = 0.011) and visiting a health facility twice or more in previous year (aPR = 1.3; 95% CI: 1.2, 1.5; *p*<0.001) were independently associated with uptake of HIV testing.

**Conclusion:**

Despite high PITC uptake, about 2 in 5 men attending healthcare facilities in Moshi municipality were not offered the service. Providers should target men aged ≤ 34 years, with primary education, visiting facilities for the first time and who have never been tested for HIV.

## Introduction

HIV Testing and Counselling (HTC) is a critical entry-point for care and treatment as well as primary and secondary HIV prevention efforts [1, 2]. The global burden of HIV is high in sub-Saharan Africa countries (SSA) with 69% of all People Living with HIV (PLHIV) [3]. Globally, over 9 million people in the general population (25%) are unaware of their HIV status [3]. In SSA, nearly half of the people living with HIV (45%) are unaware of their HIV status [3]. In Tanzania, it is estimated that 48% of PLHIV are unaware of their HIV status, with more males (55%) than females (38%) not knowing their HIV status [4]. Awareness of HIV status is critical in HIV diagnosis and linkage to appropriate medical care during the early stages of the disease and it has substantial clinical and public health benefits.

In 2007, WHO/UNAIDS recommended that countries with generalized HIV epidemics should adopt a policy of Provider Initiated HIV Testing and Counselling (PITC) in clinical settings, to complement voluntary HIV counselling and testing, so as to increase the number of people who are diagnosed early for HIV, started on ART and enrolled in other care and support services [5]. Provider-initiated HIV testing and counselling (PITC) was introduced in Tanzania in 2007 and adopted as recommended by the WHO with the aim of increasing early HIV diagnosis and timely access to care, treatment, and support services.

PITC refers to HIV testing and counselling which is recommended by health care providers to persons attending health care facilities as a standard component of medical care [5]. It is routine HIV counseling and testing offered to adult clients attending healthcare facilities. PITC is based on the principle of the “opt-out” approach including simplified pretest information. The client is provided with basic information to enable him/her to make an informed choice about whether to have an HIV test or not [5]. PITC is opportunistic screening, aiming to increase the number of people learning of their HIV status while receiving routine care and it is an important step towards achieving the 1^st^ 95 target of the 95-95-95 UNAIDS global goals by 2030 [6]. Tanzania has adopted an HIV test-and-treat strategy to improve timely treatment uptake among PLHIV.

In 2017, PITC uptake in Tanzania was estimated to be 52% of PLHIV aged 15–64 years, with 45% among males [7]. The national target was 60% by 2020 and 80% by 2025 within the general population [8–10]. The Tanzania Health Sector Strategic Plan HSSP V has an ambitious goal of having 95% of PLHIV know their status by 2025 [10]. Studies and national reports have shown that fewer men (47%) than women (62%) report to have ever been tested for HIV and received their results, similar to observation from other African countries [5, 11–13]. HIV-testing during pregnancy may have contributed to a higher percentage of women knowing their status than men [12]. Consequently, men are more likely to start ART at a later stage of HIV infection and thus experience higher morbidity and mortality [4]. Furthermore, lack of knowledge of HIV status results in potential for longer duration of onward transmission [14].

The Ministry of health has implemented several interventions to improve HIV testing uptake through PITC programs. The interventions include the provision of standard operating procedures, training guidelines and development of protocols for PITC, training of health care providers including on job training and seminars, supportive supervision, and quality assurance for PITC. And finally, promotion of PITC services at all healthcare facilities and community levels through outreach services by healthcare providers and trained lay PITC providers. This is a crucial step towards increasing PITC uptake in Tanzania hence strengthening preventive initiatives for HIV/AIDS [6, 15].

In the country, PITC uptake remains low, lagging behind the national target [11]. There is limited information describing PITC rates and subsequent uptake among men. This study aimed at determining the proportion and factors associated with being offered PITC and accepting the offer of PITC among men attending healthcare facilities in Moshi municipality, Kilimanjaro region.

## Methods

### Study design and settings

This was a facility-based cross-sectional study conducted in July 2019 in Moshi Municipality. Moshi municipality is among the seven districts of the Kilimanjaro region in northern Tanzania. It is the capital of the Kilimanjaro region. The municipality has an estimated population of 184,292, where men aged 15–64 years account for 48.4% of the total population [16]. The population engage in various economic activities such as business, agriculture and tourism activities. The prevalence rate of HIV in Kilimanjaro region is 2.6% [7]. There are 57 healthcare facilities in Moshi, half of them are public facilities. All facilities provide HIV counselling and testing free of charge.

### Study population

Men aged 18 years and above, attending outpatient clinics at five selected public healthcare facilities in Moshi municipality were invited to participate in the study.

### Sample size and sampling

The minimum sample size for men clients was obtained using the formula for single proportion. The prevalence of PITC used in the calculation was 8% [11], margin of error was set at 5% and standard normal deviation offset at 1.96. Accounting 10% non-response, the minimum sample was estimated to be 502 men.

A two-stage sampling technique was used to select the participants. The first stage involved the selection of healthcare facilities. Three hospitals; a zonal (Kilimanjaro Christian Medical Centre (KCMC)), regional (Mawenzi Regional Hospital) and district hospital (St. Joseph hospital) were purposively selected. These three facilities were selected because they have the highest number of attendees in their outpatient department (OPD) within the municipality. In addition, two (Pasua Health Centre and Bondeni Dispensary) out of 23 primary health care facilities in the municipal were randomly selected from the list of all primary public healthcare facilities.

The second stage involved the selection of clients. Using a probability proportionate to size (PPS) method the required numbers of OPD male clients were selected from each level of the five selected public healthcare facility which offers PITC services. A total of 245 clients were required from KCMC, 95 from Mawenzi Regional Referral Hospital, 91 from St. Joseph District Designed Hospital, 58 from Pasua Health Centre and 13 from Bondeni Dispensary. The required number of men clients in a particular facility was systematically selected from the OPD. Clients were approached after completing medical services by researchers for interviews.

### Study procedures

Seven research assistants with medical background (nurses and final year medical students) were selected and one day of training was conducted on the data collection tool, data collection methods and procedures, after explaining the objectives of the study, before starting data collection procedures. A brief presentation on research ethics was incorporated into this training.

A pilot study was conducted before the actual data collection at one health care facility in Moshi Municipality which was not among the selected facilities, for pretesting the data collection tool.

The initial screening for eligible clients was done by a research assistant at the outpatient department. Each man leaving the clinical room after completing medical services was informed about the study and asked if they were interested in participating in the study. Exit interviews were conducted in a private room/space located within the study outpatient department, taking an average of 10–15 minutes.

The interviewers provided detailed descriptions of the study, including the purpose of the study, assurance of confidentiality of the information provided and benefits for participating. Those who were willing to participate were asked to sign an informed consent before the interview. Clients who declined participation were not included and were assured of continued medical services Quality checks were conducted daily after completing field activities.

### Statistical analysis

Data cleaning and analysis was performed using Statistical Package for Social Sciences (SPSS) (IBM Corp. Released 2017. IBM SPSS Statistics for Windows, Version 25.0. Armonk, NY: IBM Corp). Cleaning of data was done to check for inconsistencies, missing data, recoding and categorization of variables. Categorical data was summarised using frequencies and percentages while measures of central tendency with their corresponding measures of dispersion were used to summarise numerical data.

Since the study outcome was common in this population, logistic regression analysis was not appropriate for modelling odds of experiencing the outcome of interest as it would have overestimated the odds ratio. To overcome this, log binomial regression was applied as an alternative method, but the challenge of convergence was experienced during multivariate analysis. Finally, Modified Poisson regression with robust standard error was applied to estimate crude and adjusted prevalence ratios (PR). All variables found to be significant at p-value < 0.05 in the bivariate analysis and those considered as potential confounders were included in the multivariate analysis.

### Ethical approval

The Kilimanjaro Christian Medical University College Research and Ethical Review Committee reviewed and approved the study (PG.002). Moshi Municipality Authorities gave approval to conduct the study at government healthcare facilities. Written informed consent was obtained from each participant before the interviews.

## Results

### Enrolment summary

A total of 600 men aged 18 years and above that attended the 5 participating health facilities in Moshi Municipal Council were approached to participate in the study. Of the 600, 562 consented to take part in this study, giving a response rate of 93.7%. Of the 38 participants who did not give consent to participate, the main reasons were: 18 (47.4%) limited time, 10 (26.3%) tired and 10 (26.3%) had no reasons for participating.

### Background characteristics of the participants

In total, 562 adult men were enrolled in this study. The median age of participants at enrollment was 37 (Interquartile range, 26–59) years. Most participants had secondary or higher education (58%); were married (60.7%) and residing in urban areas (62.5%). Table 1 shows background characteristics of the participants.

**Table 1:** Background characteristics of the study participants (N = 562).

Characteristics	n (%)
**Age (years)**	
18–34	267 (47.5)
35–59	166 (29.5)
60+	129 (23.0)
*Median (IQR)*	*37 (26–59)*
**Education level**	
None	49 (8.7)
Primary	190 (33.8)
Secondary	168 (29.9)
Higher education	155 (57.6)
**Marital status**	
Married	341 (60.7)
Single	184 (32.7)
Divorced/widowed/separated	37 (6.6)
**Place of residence**	
Urban	351 (62.5)
Rural	211 (37.5)

### Offer and uptake of PITC

Of the 562 adult men enrolled in this study, 324 (58%) reported to have been offered PITC on the day of the interview. Most men (83%) accepted the offer of an HIV test. The highest proportion of men who accepted HIV-testing after being offered PITC were; married (84.5%) or single (85.1%); with one sexual partner (87.4%); had ever heard about HIV (83.5%); had ever been tested for HIV (86.0%); had previously visited the health facility where they were recruited in the study (90.2%); and had a positive attitude towards PITC (83.3%). Table 2 depicts these results.

**Table 2:** Proportion of men who reported being offered PITC and accepted the offer of a test (N = 562).

Variable	Total N	Offered PITC n (%)	Accepted PITC offer n (%*)
**Age**			
18–34	267	144 (53.9)	122 (84.7)
35–59	166	107 (64.5)	84 (78.5)
60+	129	73 (56.6)	62 (84.9)
**Education level**			
None	49	35 (71.4)	27 (77.1)
Primary	190	101 (53.2)	86 (85.1)
Secondary	168	92 (54.8)	69 (75.0)
Post-secondary	155	96 (61.9)	86 (89.6)
**Marital status**			
Single	184	101 (54.9)	86 (85.1)
Currently in union	341	200 (58.7)	169 (84.5)
Formerly in union	37	23 (62.2)	13 (56.5)
**Place of residence**			
Urban	351	208 (59.3)	169 (81.3)
Rural	211	116 (55)	99 (85.3)
**Number of current sexual partners**			
None	30	15 (50.0)	10 (66.7)
One	367	223 (60.8)	195 (87.4)
Multiple	147	76 (51.7)	57 (75.0)
**Ever heard about HIV**			
No	6	3 (50.0)	0 (0.0)
Yes	556	321 (57.7)	268 (83.5)
**Ever tested for HIV**			
No	109	52 (47.7)	34 (65.4)
Yes	453	272 (60.0)	234 (86.0)
**Attitude towards PITC**			
Negative	12	7 (58.3)	4 (57.1)
Positive	550	317 (57.6)	264 (83.3)
**First visit to this facility**			
No	370	214 (57.8)	193 (90.2)
Yes	192	110 (57.3)	75 (68.2)
**Attended any health facility in the past 12 months**			
No	117	73 (62.4)	58 (79.5)
Yes	445	250 (56.3)	210 (84.0)

*Calculated out of those who were offered PITC

### Factors associated with being offered PITC

Table 3 shows factors associated with being offered PITC among the study participants. In a multivariate analysis, age between 35–59 years (aPR = 1.2; 95% CI: 1.0, 1.4; *p* = 0.033) and having primary education (aPR = 0.7; 95% CI: 0.6, 0.9; *p* = 0.010) were independently associated with being offered PITC.

**Table 3:** Factors associated with being offered PITC among study participants (N = 562).

Factor	% Offered PITC	Crude		Adjusted	
		PR (95%CI)	*p*-value	aPR (95%CI)	*p*-value
**Age**					
18–34	53.9	1		1	
35–59	64.5	1.2 (1.0,1.4)	0.027	1.2 (1.0, 1.4)	0.033
60+	56.6	1.0 (0.9,1.3)	0.615	1.0 (0.9,1.3)	0.721
**Education level**					
None	71.4	1		1	
Primary	53.2	0.7 (0.6, 0.9)	0.009	0.7 (0.6, 0.9)	0.010
Secondary	54.8	0.8 (0.6, 0.9)	0.020	0.8 (0.6, 1.0)	0.063
Post-secondary	61.9	0.9 (0.7, 1.1)	0.196	0.9 (0.7, 1.1)	0.291
**Marital status**					
Single	54.9	1			
Currently in union	58.7	1.1 (0.9, 1.3)	0.413		
Formerly in union	62.2	1.1 (0.9, 1.5)	0.390		
**Place of residence**					
Urban	59.3	1			
Rural	55.0	0.9 (0.8, 1.1)	0.327		
**Number of sexual partners**					
None	50.0	1			
One	60.8	1.2 (0.8, 1.8)	0.298		
Multiple	51.7	1.0 (0.7, 1.5)	0.867		
**Ever tested for HIV**					
No	47.7	1		1	
Yes	60.0	1.3 (1.0, 1.6)	0.032	1.2 (1.0, 1.5)	0.060
**Ever heard about HIV**					
No	47.7	1			
Yes	60.0	1.2 (0.5, 2.6)	0.726		
**First visit to this facility**					
No	57.8	1			
Yes	57.3	1.0 (0.9, 1.2)	0.843		
**Attended any health facility in the past 12 months**					
No	62.4	1			
Yes	56.3	0.9 (0.8. 1.1)	0.217		
**Attitude towards PITC**					
Negative	58.3	1			
Positive	57.6	1.0 (0.6, 1.6)	0.961		

PR-Prevalence Ratio, aPR-Adjusted Prevalence Ratio; CI-Confidence Interval

### Factors associated with PITC uptake

Table 4 presents factors associated with accepting the PITC offer among study participants. In a multivariate analysis, age between 35–59 years (aPR = 0.8; 95% CI: 0.7, 0.9; *p* = 0.002); ever tested HIV (aPR = 1.3; 95% CI: 1.1, 1.5; *p* = 0.011) and not being a first visit to the health facility where the client was enrolled (aPR = 1.3; 95% CI: 1.2, 1.5; *p*<0.001) were independently associated with accepting the offer of PITC.

**Table 4:** Factors associated with PITC uptake among study participants (N = 324).

Factor	% Uptake PITC	Crude		Adjusted	
		PR (95%CI)	*p*-value	aPR (95%CI)	*p*-value
**Age**					
18–34	84.7	1		1	
35–59	78.5	0.9 (0.8, 1.0)	0.218	0.8 (0.7, 0.9)	0.002
60+	84.9	1.0 (0.9, 1.1)	0.968	0.9 (0.8, 1.0)	0.073
**Education level**					
None	77.1	1		1	
Primary	85.1	1.1 (0.9, 1.3)	0.329	1.0 (0.8, 1.2)	0.876
Secondary	75.0	1.0 (0.8, 1.2)	0.798	0.9 (0.7, 1.1)	0.158
Post-secondary	89.6	1.2 (1.0, 1.4)	0.129	1.0 (0.8, 1.2)	0.746
**Marital status**					
Single	84.5	1			
Currently in union	85.1	1.0 (0.9, 1.1)	0.882		
Formerly in union	56.5	0.7 (0.5, 0.9)	0.030		
**Place of residence**					
Urban	81.3	1			
Rural	85.3	1.1 (1.0, 1.2)	0.950		
**Number of sexual partners**					
None	66.7	1		1	
One	87.4	1.3 (0.9, 1.9)	0.142	1.4 (1.0, 2.0)	0.054
Multiple	75.0	1.1 (0.8, 1.6)	0.545	1.2 (0.8, 1.7)	0.332
**Ever tested for HIV**					
No	65.4	1		1	
Yes	86.0	1.3 (1.1, 1.6)	0.008	1.3 (1.1, 1.5)	0.011
**First visit to this facility**					
No	90.2	1.3 (1.2, 1.5)	<0.001	1.3 (1.2, 1.5)	<0.001
Yes	68.2	1		1	
**Attended any health facility in the past 12 months**					
No	79.5	1			
Yes	84.0	1.1 (0.9, 1.2)	0.397		
**Attitude towards PITC**					
Negative	57.1	1			
Positive	83.3	1.5 (0.8, 2.8)	0.252		

PR-Prevalence Ratio, aPR-Adjusted Prevalence Ratio; CI-Confidence Interval

### Reasons for not accepting the PITC offer

**Fig 1:**
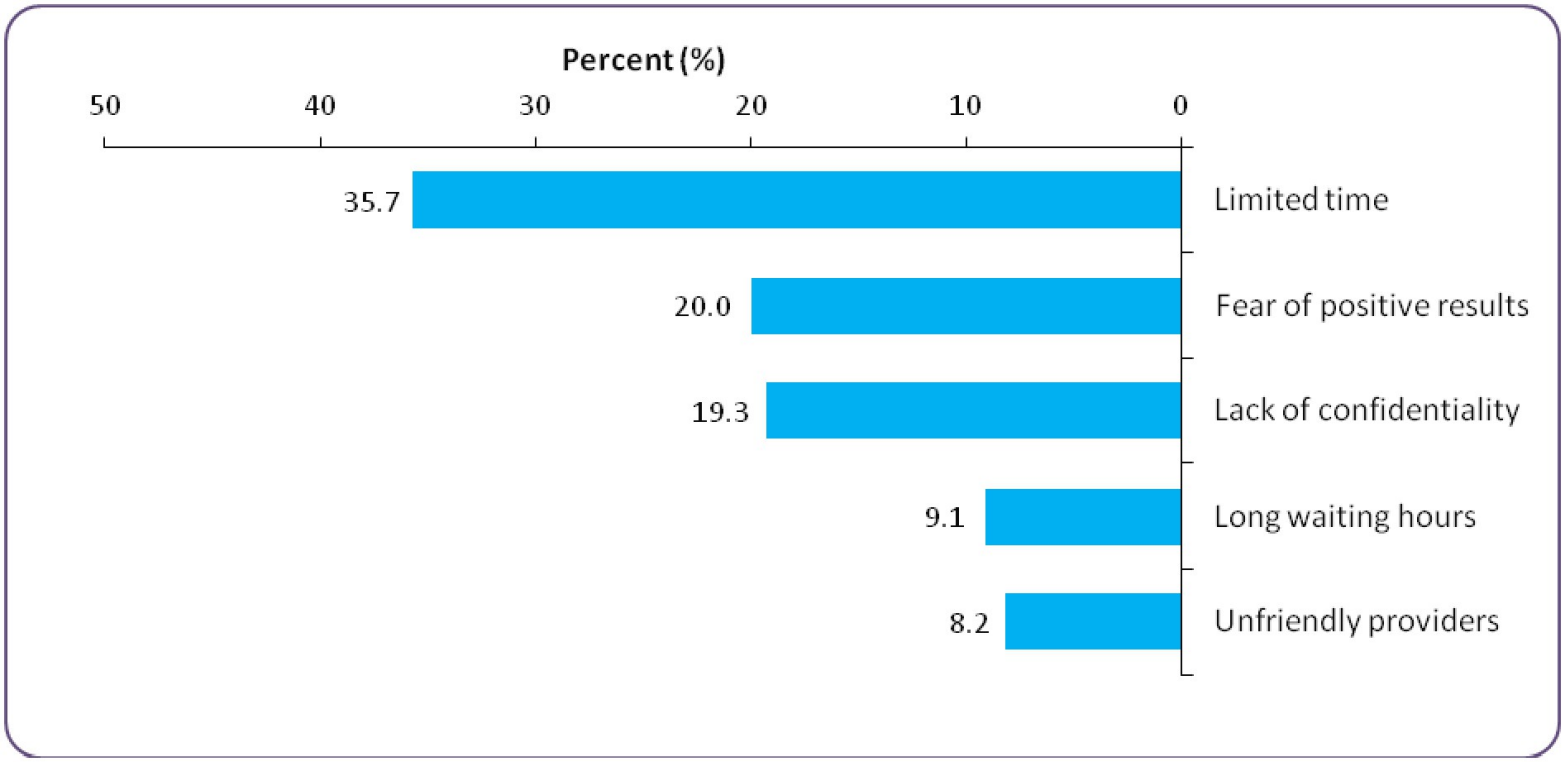
Reasons for not accepting the PITC offer (n = 55).

Fig 1 presents reasons for not accepting the PITC offer. Among 55 participants who did not accept the PITC offer; the main reasons included no time for the test (35.7%), fear of positive test results (20.0%) and lack of confidentiality (19.3%).

## Discussion

Six out of every 10 men enrolled in the study were offered PITC, and of those offered, 83% completed an HIV test. The proportion of men who reported to have ever been tested for HIV were high (81%). The factors which were found to be significantly associated with being offered HIV testing and counselling among study participants were age and number of sexual partners. Having been tested for HIV, first visit to the facility and walking distance to the nearest facility were the main independent predictors which were significantly associated with PITC uptake.

Nearly half (42%) of men who attended healthcare services were not offered an HIV test by their healthcare provider. This implies that there is a high proportion of missed opportunities by providers at these facilities for not screening these men. The missed opportunities include early detection of HIV infection, timely referral for entry into Counselling and Treatment centres for those diagnosed HIV positive and for Antiretroviral Therapy (ART) initiation, other care and support services. Our findings are comparable to other studies in the region (36% in Kenya, 35% in Ethiopia) [17, 18]. The prevalence of PITC uptake in our study is high. This corresponds with research done in Ethiopia (72.3%), SSA (85%) and several developed countries [19, 20]. This is the case in most healthcare facilities in many countries according to the literature. This may be due to effectiveness of pre-test and post-test counselling by providers which provides men with sufficient information on the importance of knowing their HIV status. This is important to reduce morbidity and mortality to Men living with HIV (MLWH) as it enables them to get ART early and engage in secondary preventive measures.

According to our study results, age of participants was statistically significant associated with being offered HIV testing and counselling. Men aged 35–59 years were more likely to be offered PITC than both older and younger men. Similar findings reported in South Africa and Burkina Faso [21, 22] revealed that older age had been associated with being offered HIV testing and counselling more than with younger age. Young men, however, also need interventions designed to improve HIV testing uptake, since these men are less likely to present at conventional health facilities. Having two or more sexual partners was significantly associated with an 80% lower chance of being offered PITC compared to those with no or only a single sexual partner. Similar findings were observed in South Africa [21]. A consequence of not offering PITC services to this risk group for HIV/AIDS acquisition, is the risk of greater progressive HIV transmission within the general population.

Having been tested for HIV significantly associated with 1.3 times greater PITC uptake than for those never previously tested for HIV. Similar findings have been reported in Zambia and New York [23, 24]. It may be possible that most of these men have frequent contact with healthcare facilities which might provide adequate information on HIV testing and counselling. In addition, they might know their HIV status, which is why they accept PITC more readily than those never tested for HIV.

Limited time, fear of positive results and lack of confidentiality were the most reported reasons for not accepting HIV testing among those participants who did not accept PITC. Similar results have been observed elsewhere [25, 26]. This implies that there is a need to broaden the modality in which PITC services for men in our health facilities is practiced. It should motivate more men to accept PITC.

The major strength of this study includes involvement of all levels of healthcare facilities with a large sample size allowing generalizability of the study results. The key limitations of this study include its cross-sectional nature thus causal relationship could not be established. The key outcome variable i.e., the uptake of PITC, was gathered by self-report. Reporting bias may have overestimated the observed results. Clients who were admitted after the OPD consultations were missed in the exit interviews. Lastly, information from providers was not collected. This may have confirmed the reports from the men. This needs to be done in future studies.

## Conclusions

Despite the high uptake of PITC, more than one third (42.3%) of the men were not offered PITC. The HIV testing and counselling offered was found to be significantly associated with age and number of sexual partners. The history of having been tested for HIV and walking distance to the nearest facility were significantly associated with PITC uptake. Strategies to improve PITC in this setting are needed, including provider training and re-training on guidelines as it has been shown to be effective in diagnosing more than 70% of all new HIV cases among men and women in another region in Tanzania [27]. Optimizing the offer of PITC to men at every encounter is important given that 9 out of 10 will accept testing. Enhancing targeted testing at other service delivery points such as TB clinics, STI clinics, cancer screening clinics or others depending on the setting, may improve detection of new HIV cases [12, 28]. In this setting, prioritising PITC for males who have never been tested for HIV, who are younger than 35 years and who have higher education may increase the number tested.

Further research to assess the offer of PITC services among providers as well as health system factors influencing the offer for PITC services among men should be conducted.

## Supporting information

S1 Appendix Probability proportionate to size sampling for the 5 public healthcare facilities offering PITC in Moshi Municipality-Kilimanjaro region. (DOCX)

S2 Appendix Male client’s questionnaire (Exit interview). (DOCX)

S3 Appendix (XLSX)

## Abbreviations

aOR: Adjusted Odds Ratio
aPR: Adjusted Prevalence Ratio
CI: Confidence Interval
HTC: HIV Testing and Counselling
KCMC: Kilimanjaro Christian Medical Centre
OPD: Outpatient Department
OR: Odds Ratio
PITC: Provider Initiated HIV Testing and Counselling
PR: Prevalence Ratio
SSA: Sub-Saharan Africa
WHO: World Health Organization
