# Supplementary material for: Factors associated with offer and uptake of provider-initiated HIV testing and counselling among men attending healthcare facilities in Moshi Municipality, Northern Tanzania
Source: PLoS One. 2023 Sep 20;18(9):e0291792. doi: 10.1371/journal.pone.0291792 (PMC10511071; doi:10.1371/journal.pone.0291792)
Supplement: S1 Appendix — (DOCX) [file pone.0291792.s001.docx]

**APPENDIX**

**Table 1: Probability proportionate to size sampling for the 5 public healthcare facilities offering PITC in Moshi Municipality-Kilimanjaro region.**

| **Name of Healthcare Facility** | **Estimate Number of male clients attending OPD per week** | **Proportion contribution per each facility** | **Estimated male sample required per facility** |
| --- | --- | --- | --- |
| KCMC Referral Hospital | 1,340 | 0.489 | 245 |
| Mawenzi Regional Hospital | 520 | 0.189 | 95 |
| St. Joseph Hospital | 500 | 0.182 | 91 |
| Pasua Health centre | 313 | 0.114 | 58 |
| Bondeni Dispensary | 68 | 0.025 | 13 |
| Total | **2,741** | **1.0** | **502** |
