# Supplementary material for: Factors associated with offer and uptake of provider-initiated HIV testing and counselling among men attending healthcare facilities in Moshi Municipality, Northern Tanzania
Source: PLoS One. 2023 Sep 20;18(9):e0291792. doi: 10.1371/journal.pone.0291792 (PMC10511071; doi:10.1371/journal.pone.0291792)
Supplement: S2 Appendix — (DOCX) [file pone.0291792.s002.docx]

**Appendix 2A: Male Client’s Questionnaire (Exit Interview)**

**QUESTIONAIRE**

Serial No. **[__|__|__|__]**

**QUESTIONNAIRE FOR OUTPATIENT MEN CLIENTS**.

|  | | **GENERAL INFORMATION** | | | |  |
| --- | --- | --- | --- | --- | --- | --- |
| **ID of participant**  …………………………. | | **Date of interview**  ………………………………… | | **Name of interviewer:**  ……………………………… | |  |
|  | | **Name of district**  …………………………………. | | **Department**  ……………………………… | |  |
|  | |  | |  | |  |
| **SN** | **QUESTIONS** | | **RESPONSES** | | **CODES** | |
|  | **SOCIO – DEMOGRAPHIC CHARACTERISTICS** | | | |  | |
| 1 | (a) Date of birth | | DD/MM/YYYY  [________] | |  | |
|  | (b)Age | | ………….. years | |  | |
| 3 | Marital status | | 1. Married 2. Cohabiting 3. Single 4. Divorced/separated/widowed | | 1  2  3  4 | |
| 4 | Occupation | | 1. Employed 2. Self employed 3. Unemployed 4. Student | | 1  2  3  4 | |
| 5 | Place of residence | | 1. Urban 2. Rural | | 1  2 | |
| 6 | What is your income per month (in Tsh)? | | 1. ≤ 50,000 2. > 50,000 | | 1  2 | |
|  | **QUESTIONS PERTAINING TO RISK** **SEXUAL BEHAVIOURS** | | | |  | |
| 7 | How many sexual partners do you have? | | …………………………. | |  | |
| 8 | Have you had sexual intercourse during the last three months? | | 1. Yes 2. No *skip to 11* | | 1  0 | |
| 9 | How many different partners have you had sexual intercourse with during the last three months? | | 1. More than one  2. Only one  3. None | | 1  2  3 | |
| 10 | Thinking about all the times you had sexual intercourse with any partner during the last sex in the past three months, would you say that you and your partner[s] used condom? | | 1. Yes 2. No | | 1  0 | |
|  | **QUESTIONS PERTAINING TO ACCESS AND USE OF HEALTH FACILITY** | | | |  | |
| 11 | How long would it take you to get to the nearest health facility that offers HIV testing and counselling from your home? | | …………………………… | |  | |
| 12 | Is this your first visit to this facility? | | 1.Yes  0. No | | 1  0 | |
| 13 | Apart from today, have you attended this facility for services in the past one year? | | 1. Yes  0. No | | 1  0 | |
| 14 | Have you attended at any other healthcare facility in the past one year? | | 1. Yes  0. No *skip to 16* | | 1  0 | |
| 15 | What type of services have you received in previous visits? | | 1. Health education  2. Medical examination  3. Medicines  4. Surgery  5. Others (Specify)…………………. | | 1  2  3  4  5 | |

|  | **QUESTIONS ON HIV TESTING HISTORY** | | | | |  | |
| --- | --- | --- | --- | --- | --- | --- | --- |
| 16 | Have you ever heard about HIV?  *(Not under PITC setting)* | 1. Yes  0. No | | | | 1  0 | |
| 17 | Have you ever been tested for HIV?  *(Not under PITC setting)* | 1. Yes  0. No *skip to 24* | | | | 1  0 | |
| 18 | Do you know where to get HIV testing services? | 1.Yes  0. No. | | | | 1  0 | |
| 19 | If Yes, which place do you mean? | 1. At health care or testing facility  2. At home  3. HIV Testing Services Campaigns  4. Other, please specify ......... | | | | 1  2  3  4 | |
| 20 | If Yes in **# 17** When was the last time you tested (mm/yyyy)? | ……………………………………….. | | | |  | |
| 21 | How many times have you been tested for HIV? | …………………. (number of times tested) | | | |  | |
| 22 | What was the reason for you having a test? | ………………………………………….. | | | |  | |
|  |  |  | | | |  |  |
| 23 | Do you feel comfortable disclosing your HIV status? | 1. Yes 2. No | | | | 1  0 | |
| 24 | If, no what are the reasons? | 1. I would be treated badly by health workers. | | | 1.Yes  0.No | 1  0 | |
|  |  | 1. I would lose my job, customers, or livelihood. | | | 1.Yes  0.No | 1  0 | |
|  |  | 1. My family would not take care of me when I am sick. | | | 1.Yes  0.No | 1  0 | |
|  |  | 1. People would gossip about me. | | | 1.Yes  0.No | 1  0 | |
|  |  | 1. I would not be allowed to participate fully within the community. | | | 1.Yes  0.No | 1  0 | |
|  |  | 1. Others (Specify)…………………. | | |  |  | |
| 26 | If not tested, what were the reasons | 1. Not counselled | | 1. Yes  0. No | | 1  0 | |
|  |  | 1. Fear of positive results | | 1. Yes  0. No | | 1  0 | |
|  |  | 1. Stigma | | 1. Yes  0. No | | 1  0 | |
|  |  | 1. Limited time | | 1. Yes  0. No | | 1  0 | |
|  |  | 1. Lack of confidentiality | | 1. Yes  0. No | | 1  0 | |
|  |  | 1. Unfriendly providers | | 1. Yes  0. No | | 1  0 | |
|  |  | 1. Long waiting hours | | 1. Yes  0. No | | 1  0 | |
|  |  | 1. Others (Specify) | |  | |  | |
|  | **QUESTIONS ON AWARENESS, ATTITUDE AND PITC UPTAKE** | | | | |  | |
| 27 | Have you ever heard about PITC before? | 1. Yes 2. No *skip to 29* | | | | 1  0 | |
| 28 | If yes, what was the source of your information? | 1. Media  2. Meetings/seminars  3. Healthcare providers  4. Friends  5. Relatives/family members | | | | 1  2  3  4  5 | |
| 29 | What health problem are you suffering from today? | …………………………………….. | | | |  | |
| 30 | For how long have you stayed with the health problem you have mentioned above? | …………………………………….. | | | |  | |
| 31 | Which services did you receive today? | 1. Health education 2. Investigations 3. Medicines 4. Minor surgery 5. Others (Specify)…………………. | | | | 1  2  3  4  5 | |
| 32 | Apart from services which you came for, did any health provider talk about HIV testing services? | 1. Yes 2. No | | | | 1  0 | |
| 33 | Apart from services which you came for TODAY, did any health provider **OFFER YOU** an HIV Testing? | 1. Yes 2. No *skip to 37* | | | | 1  0 | |
| 34 | Which staff offered you an HIV test?  (**Read list and check all respondent mentioned**) | 1. Clinician 2. Nurse 3. Nursing supervisor 4. Midwife 5. Lab technician | | | | 1  2  3  4  5 | |
| 35 | Did you **ACCEPT** the offer for an HIV test? | | 1. Yes  2. No | | | | 1  0 |
| 36 | If no, what are the reasons for you not accepting the offer for HIV testing? | | ……………………………………………… | | | |  |
| 37 | If you were offered HIV counselling and testing today, would you had accepted and test for HIV? | | 1. Yes  2. No | | | | 1  0 |
| 38 | If no, why? | | ………………………………………………. | | | |  |
| 39a | Do you think that, it is a good practice of offering men HIV testing and counselling, at a healthcare facility by a provider, when they come for something else? | | 1. Strongly disagree  2. Disagree  3. Agree  4. Strongly agree | | | | 1  2  3  4 |
| 39 b | Do you think it is important for the providers, to ensure that people know their HIV status? | | 1. Strongly disagree  2. Disagree  3. Agree  4. Strongly agree | | | | 1  2  3  4 |
| 39 c | Do you think that it is okay for healthcare provider to require certain people to have an HIV test? | | 1. Strongly disagree  2. Disagree  3. Agree  4. Strongly agree | | | | 1  2  3  4 |
| 39 d | Do you think that PITC makes easier for clients to get tested? | | 1. Strongly disagree  2. Disagree  3. Agree  4. Strongly agree | | | | 1  2  3  4 |
| 39 e | Do you feel that PITC results in less stigma and discrimination of HIV positive patients? | | 1. Strongly disagree  2. Disagree  3. Agree  4. Strongly agree | | | | 1  2  3  4 |
|  | **SUGGESTIONS** | | | | | |  |
| 40 | What are you suggesting on how to improve PITC services?  *(Don’t exceed the space provided)* | |  | | | |  |

**THANK YOU FOR YOUR PARTICIPATION.**
